# Gene Transfer and Genome-Wide Insertional Mutagenesis by Retroviral Transduction in Fish Stem Cells

**DOI:** 10.1371/journal.pone.0127961

**Published:** 2015-06-01

**Authors:** Qizhi Liu, Yunzhi Wang, Fan Lin, Lei Zhang, Yan Li, Ruowen Ge, Yunhan Hong

**Affiliations:** Department of Biological Sciences, National University of Singapore, Singapore, Singapore; Leibniz Institute for Age Research - Fritz Lipmann Institute (FLI), GERMANY

## Abstract

Retrovirus (RV) is efficient for gene transfer and integration in dividing cells of diverse organisms. RV provides a powerful tool for insertional mutagenesis (IM) to identify and functionally analyze genes essential for normal and pathological processes. Here we report RV-mediated gene transfer and genome-wide IM in fish stem cells from medaka and zebrafish. Three RVs were produced for fish cell transduction: rvLegfp and rvLcherry produce green fluorescent protein (GFP) and mCherry fluorescent protein respectively under control of human cytomegalovirus immediate early promoter upon any chromosomal integration, whereas rvGTgfp contains a splicing acceptor and expresses GFP only upon gene trapping (GT) via intronic in-frame integration and spliced to endogenous active genes. We show that rvLegfp and rvLcherry produce a transduction efficiency of 11~23% in medaka and zebrafish stem cell lines, which is as 30~67% efficient as the positive control in NIH/3T3. Upon co-infection with rvGTgfp and rvLcherry, GFP-positive cells were much fewer than Cherry-positive cells, consistent with rareness of productive gene trapping events versus random integration. Importantly, rvGTgfp infection in the medaka haploid embryonic stem (ES) cell line HX1 generated GTgfp insertion on all 24 chromosomes of the haploid genome. Similar to the mammalian haploid cells, these insertion events were presented predominantly in intergenic regions and introns but rarely in exons. RV-transduced HX1 retained the ES cell properties such as stable growth, embryoid body formation and pluripotency gene expression. Therefore, RV is proficient for gene transfer and IM in fish stem cells. Our results open new avenue for genome-wide IM in medaka haploid ES cells in culture.

## Introduction

Gene transfer is a routine to study the molecular mechanisms that control various processes in diverse organisms. For in vivo gene transfer into eggs and embryos, microinjection has widely been used in mouse [1] and other organisms including goldfish [2], zebrafish [3] and medaka [4–6].In vitro gene transfer into cultured cells has been achieved by chemical reagents, electroporation and baculoviral infection [7–10]. Generally, viral vectors provide higher efficiency for gene transfer [11–14] and therapy [15–20]. Among viral vectors, the pantropic retrovirus (RV) pseudotyped with the vesicular stomatitis virus G glycoprotein (VSVG) features a broad host cell range [21–24] for gene transfer in various organisms including mouse[25], zebrafish [26–30], medaka [31], live-bearing fish and crustaceans[32]. RV stably introduces transgenes into the genome of dividing cells with a high efficiency and represents a standard for insertional mutagenesis (IM) in cell cultures. RV-mediated IM in near-haploid human leukemia cell lines (near-haploid KBM7 and HAP1) has led to the identification of genes for host factors necessary for bacterial and viral infection [33–37] and for cellular phenotypes [38–40].

This study was aimed to develop and make use of RVs for gene transfer and IM in stem cell lines of medaka and zebrafish, the twin fish models for vertebrate development. Medaka has given rise to several stem cell lines including diploid embryonic stem (ES) cell lines [41] capable of chimera formation [42–44], haploid ES cell lines capable of whole animal production by semi-cloning [10, 45], a male germ stem cell line called SG3 capable of test-tube sperm production [9], and primordial germ cell cultures from midblastula embryos [46]. In zebrafish, we have also derived ES-like cells in feeder-free culture [47, 48]. Here we show that RV is able to mediate a high efficiency of gene transfer and chromosomal integration in fish stem cell cultures and more importantly, to offer proficiency for genome-wide IM in medaka haploid ES cells.

## Materials and Methods

### Plasmids

Plasmid pLegfp was purchased from Clontech, which contains retroviral elements derived from a Moloney murine leukemia virus (MoMuLV) and egfp expression cassette under control of the CV promoter. Plasmid pLcherry is a derivative of pLegfp by replacing the egfp with cherry PCR-amplified by using primers (AAAACCGGTATGGTGAGCAA and TAGTAGTGATTTAGCTAGGG) from pCS2cherry. pGTgfp and retroviral packaging plasmids: pAdvantage, pGag/pol and pVSV-G were kindly provided by Dr. Thijn R. Brummelkamp (The Netherlands Cancer Institute, Amsterdam, Netherlands) [38]. Plasmid DNA was prepared by using the Plasmid Mid-prep kit (Qiagene, Diagene, Germany).

### Cell culture

The adenovirus 5-transformed human embryonic kidney cell line 293T and mouse fibroblast cell line NIH/3T3 were obtained from ATCC and maintained in Dulbecco's modified Eagle's medium(DMEM) supplemented with 10% fetal calf serum (FCS), penicillin (100 U/ml), and streptomycin (100 μg/ml) at 37°C under 5% CO_2_. Fish cells from medaka and zebrafish were maintained in ESM4 at 37°C under ambient air as described [9, 10, 41, 42, 45]. These were the medaka diploid ES cell line (MES1) [41, 42], haploid ES cell lines (HX1 and HX2) [10], spermatogonial stem cell line (SG3) from the adult testis[9], fibroblast cells (MF) derived from 7-day-old larvae, and zebrafish ES-like cell line (Z428) derived from blastula embryos [47].

### Retrovirus production

For retroviral production, 293T cells at 80% confluence were trypsinized and single cell suspension was prepared in PBS for transfection by electroporation as described [8]. Briefly, a 400-μl mixture containing 10^7^ cells and 15 μg of plasmid DNA was electroporated with square wave delivered at 110 V and 25 ms by the Easyject electroporator (Bio-Rad). The15 μg of plasmid DNA combined 4 plasmids (1.7 μg of pAdvantage, 2.6 μg of pVSV-G, 4 μg of pGag-pol, and 6.7 μg of pLegfp, pLcherry or pGTgfp, respectively). Electroporated cells were incubated on ice for 15 min, centrifuged for 5 min at 2000 rpm (Centrifuge 5415C, Eppendorf), resuspended in DMEM-10% FCS and seeded onto 10-cm dishes for culture. The culture medium containing RVs was collected at 48h after transfection, filtrated through a 0.45-μm filter (Sartorius) and concentrated by centrifugation at 4°C for 150 min at 20,000 rpm (Beckman J2-21). The RV pellet was gently resuspended in 100-μl of ESM4 medium for storage at -80°C and cell infection.

### Cell transduction

Cells were infected with RVs by using the spinoculation procedure [37]. Briefly, 500μl of infection mixture were prepared by combining 10^6^ cells, retrovirus, polybrene (8 μg/ml, Sigma) and ESM4 medium. The mixture was spun at room temperature for 2 h at 2000 rpm (Eppendorf 5415R). The cell pellet was resuspended by gentle pipetting in100 μl of ESM4 and seeded onto 6-well plates containing 2 ml of ESM4 per well. GFP and Cherry expression were monitored by fluorescent microscopy at 3~5 day post infection (dpi) with rvLegfp or rvLcherry. Percentage of cells positive for GFP or Cherry was determined by cell counting and regarded as transduction efficiency. Retrovirus titer in different type cells was calculated with following formula and average titer was got from 3 independent infection experiments. Titer (transduction unit/ml) = [(10^6^ seeded cells x % GFP+ cells)/μl retrovirus] x 10^3^.

### Induced cell differentiation

Cells in three groups (HX1 control, HX1 transgenics and control + transgenics at a ratio of 1:1) were subjected to EB formation in suspension culture in the presence of all-trans retinoic acid (RA; 5 μM) for induced differentiation. Live EBs were stained with Hoechst 33342 (1 μg/ml). Cell differentiation was monitored by phenotype and studied by RT-PCR analyses of expression of pluripotency and lineage-specific genes [10].

### RT-PCR

Total RNA was isolated by using the Trizol Reagent (Invitrogen). Synthesis of cDNA templates was primed with oligo (dT)18 by using M-MLV transcriptase (Invitrogen). The cDNA reaction was diluted with water to 10 ng/μL. RT-PCR was run as previously described [10]. PCR primers are listed (S1 Table).

### Cell growth assay

HX1 cells infected with rvLcherry and rvGTgfp at MOI = 50 each and parental HX1 cells were analyzed for continuous growth as described [10]. Briefly, 10^5^ single cells in ESM4 were evenly distributed in 12-well plates. After 24 h of culture, non-adherent cells were removed by PBS washes and medium change, and cells were trypsinized for hemocytometric determination of the initial cell number. The medium was changed every 3 (1 ml; during first 6 days) or 2 days (2 ml; during subsequent days). Cells were counted at regular intervals until a plateau was reached.

### Linker-mediated-PCR and sequencing

DNA isolation from cell cultures was done as described [49]. Briefly, HX1 cells were harvested by trypsinization and incubated in lysis buffer (10 mM Tris-HCl, pH 8.0, 1 mM EDTA, 1% SDS, 100 mg/ml proteinase K) at 50°C for 3 h. After phenol/chloroform extraction, DNA was ethanol-precipitated and resuspended in water. The linker-mediated PCR (LM-PCR) was performed as described [38]. Briefly, MlucI digests of genomic DNA were amplified by a 5′-biotinylated primer (GGTCTCCAAATCTCGGTGGAAC) and then enriched by using the Dynabeads M280 (Invitrogen, Carlsbad, CA). The resulting single stranded DNA (ssDNA) was then ligated to a ssDNA linker (GGGAGATCTGAATTCAGTGGCACAG) by using a ssDNA ligase (CircLigase II, Epicenter Biotechnologies). After ligation, the product was purified again by using theDynabeads M280 and amplified via two rounds of PCR. The first round of PCR used primers P1 (GGGAGATCTGAATTCAGTGGCACAG) plus LTR-rev1 (ATCTGATGGTTCTCTAGCTTGCC), and the second round used P2 (AATTCAGTGGCACAG) plus LTR-rev2 (TGCCAAACCTACAGGTGGGGTCTTTCA).The PCR products were cloned into pGEM-T (Promega) and sequenced on the ABI 3130xl Genetic Analyzer. Sequence comparison and alignment were run on DNAman and Vector NTI Advance 11.5 version. Blast search was run against the medaka genome database (http://www.ensembl.org/index.html).

### Microscopy

Microscopy was performed under Zeiss Axiovert2 invert microscope equipped with a Zeiss AxioCam MRc digital camera and AxioVision 4 software.as described [10, 45].

### Statistical analysis

For statistical analyses, the Dunnett’s test was conducted by using GraphPad Prism v4.0. Data are presented as means ± SD, and P < 0.05 and P < 0.01 were considered as significant and very significant differences.

## Results

### Production and transducibility of retrovirus

**Fig 1:**
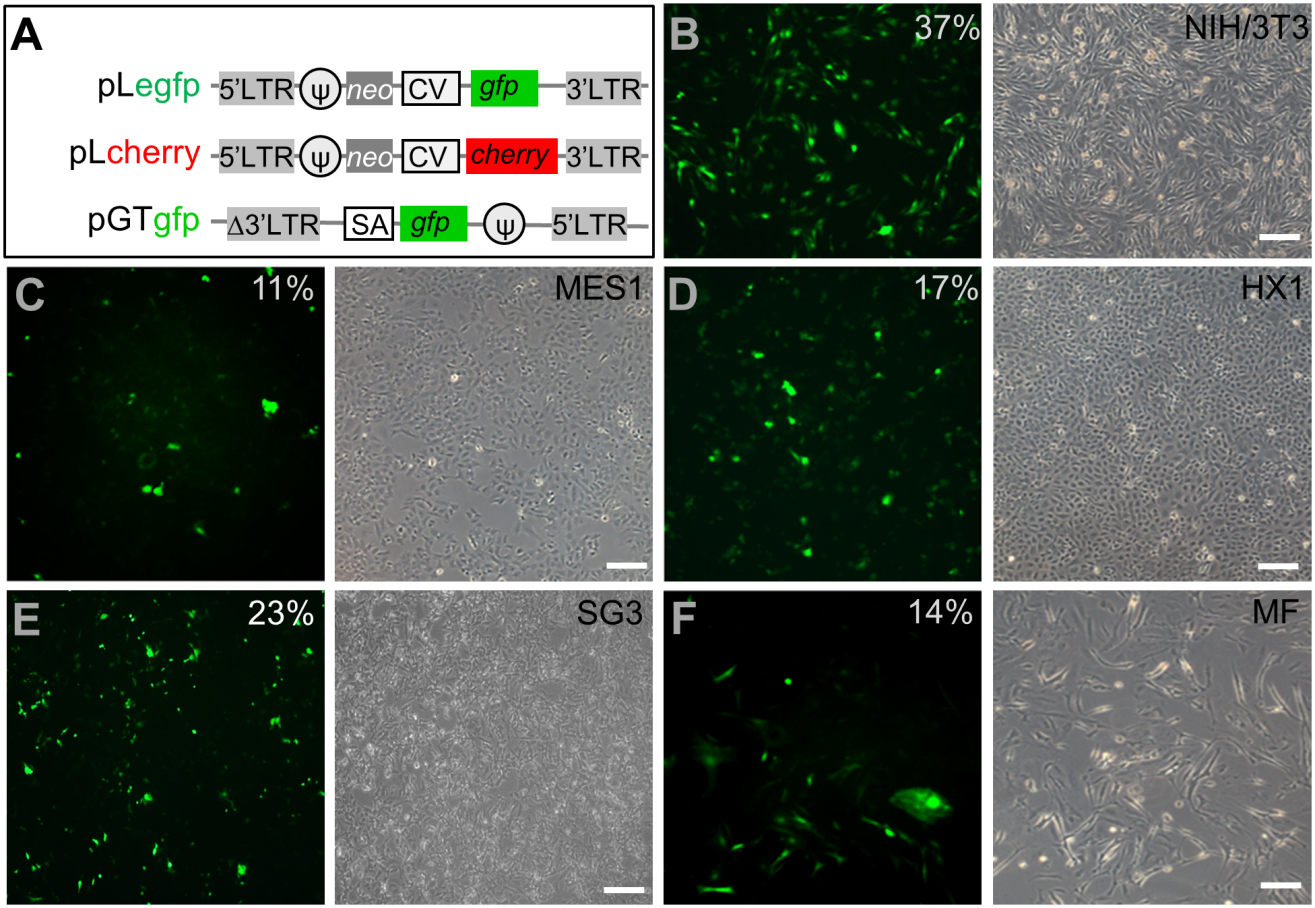
Retroviral vectors and transduction in mouse and medaka cells. (A) Maps of plasmids pLegfp, pLcherry and pGTgfp for retroviruses rvLegfp, rvLcherry and rvGTgfp. CV, human cytomegalovirus early gene enhancer/promoter; LTR, long terminal repeat; cherry, gene for cherry fluorescent protein; gfp, gene for green fluorescent protein; neo, neomycin phosphotransferase gene; psi, packaging element (ψ); SA, splicing acceptor. (B-F) Micrographs of rvLegfp-infected cells. Cells were infected at MOI = 50 and photographed at 3 dpi by using fluorescent (left panel) and phase-contrast optics (right panel). Average percentage values of GFP-positive cells (green) derived by cell counting in three independent experiments are shown. (B) NIH/3T3 as the positive control. (C) MES1. (D) HX1. (E) SG3. (F) Medaka fibroblasts in primary culture (MF). Scale bars, 100 μm.

Three retroviral plasmids were used for RV production (Fig 1A). Two of them have been used for gene transfer in a broad range of host cells: pLegfp can express GFP either transiently or stably after chromosomal integration from the strong human cytomegalovirus promoter, whereas pGTgfp will acquire the ability to stably express GFP upon a proper gene trapping event into an intron of an expressed gene. A third plasmid, pLcherry, was derived from pLegfp and able to transiently or stably express Cherry. Therefore, pLegfp and pLcherry were used to monitor gene transfer, whereas pGTgfp was used to monitor gene trapping. Each of the three plasmids was cotransfected with a set of 3 packaging plasmids in the human embryonic kidney cell line 293T for production of RVs (rvLegfp, rvLcherry and rvGTgfp, respectively). We first focused on rvLegfp for gene transfer and expression in the mouse fibroblast cell line NIH/3T3 as a positive control. At 3 dpi, in this cell line, rvLegfp produced numerous GFP-positive cells (Fig 1B), demonstrating the infectivity and transducibility of rvLegfp in mammalian cells.

### Retroviral transduction of fish stem cells

**Fig 2:**
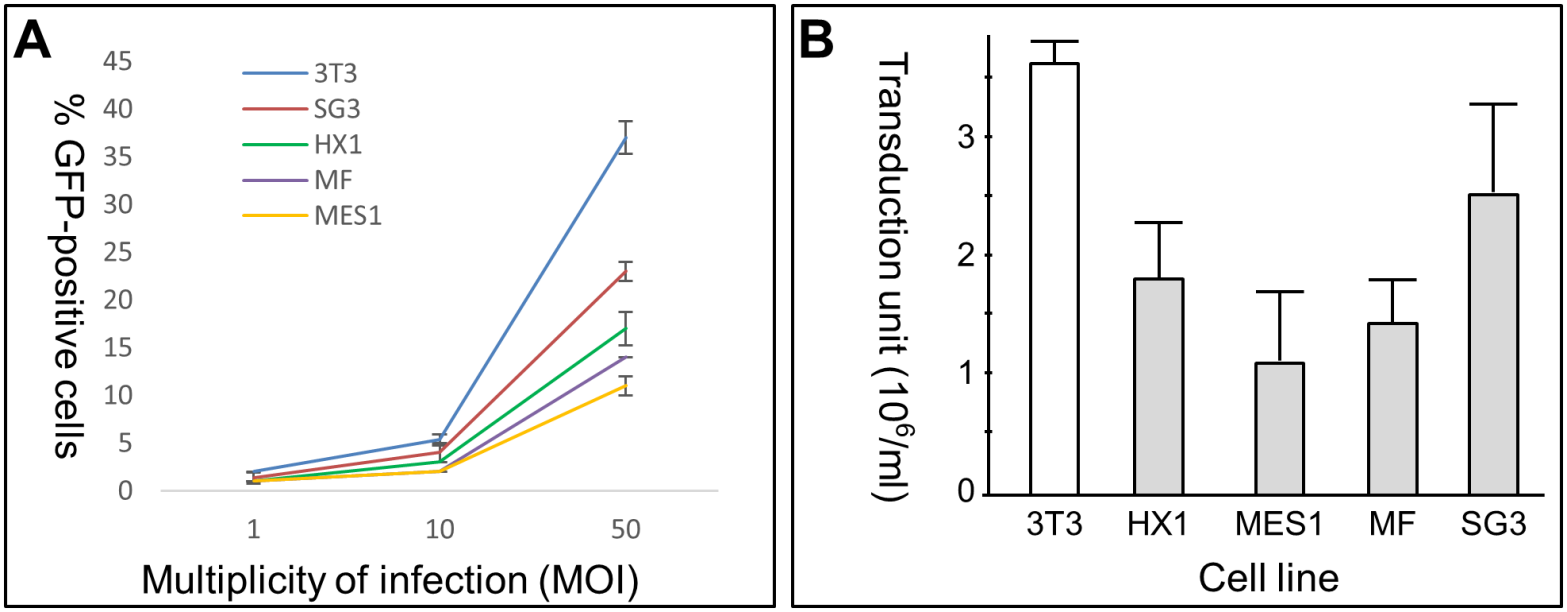
Retroviral transduction efficiency in medaka cells. (A) Dose-dependent transduction efficiency of rvLegfp in fish cell lines. Cells were infected with rvLegfp at indicated MOI and the transduction efficiency was determined by GFP expression by counting at least 1000 cells at 3 dpi. (B) Titer of rvLegfp in fish cell lines. Cells were transduced with rvLegfp at MOI of 50 and titer was determined at 3 dpi. Data are means ± SD from three independent experiments. 3T3, mouse fibroblast cell line NIH/3T3 used as the positive control; MES1, medaka diploid ES cell line; HX1, medaka haploid ES cell line; MF, medaka fibroblast cells; SG3, medaka spermatogonial cell line.

We then applied rvLegfp to stable medaka stem cell lines and primary cell culture and determined the transduction efficiency by GFP expression. The result revealed that rvLegfp was able to transduce all the medaka cells examined, including the diploid ES cell line MES1 (Fig 1C), haploid ES cell line HX1 (Fig 1D), spermatogonial stem cell line SG3 (Fig 1E) and primary fibroblast-like cell culture (Fig 1F). Cell counting at 3 dpi revealed that the transduction efficiency was dependent on multiplicity of infection (MOI) (Fig 2A). At MOI = 50, the transduction efficiency was the highest in NIH/3T3 (37%). While in medaka cells, the transduction efficiency was 11% in MES1, 17% in HX1, 23% in SG3 and 14% in primary fibroblast-like cell culture (Fig 2A). In zebrafish ES-like cell line Z428, rvLegfp under the same conditions led to a transduction efficiency of 15% (S1 Fig). A similar figure was also drawn for retroviral titer as determined as transduction unit per ml of RV-containing medium (Fig 2B). Therefore, both diploid and haploid stem cells as well as non-stem cell primary culture from medaka and ES-like cell line Z428 from zebrafish are proficient for RV-mediated gene transfer.

### Cotransduction

**Fig 3:**
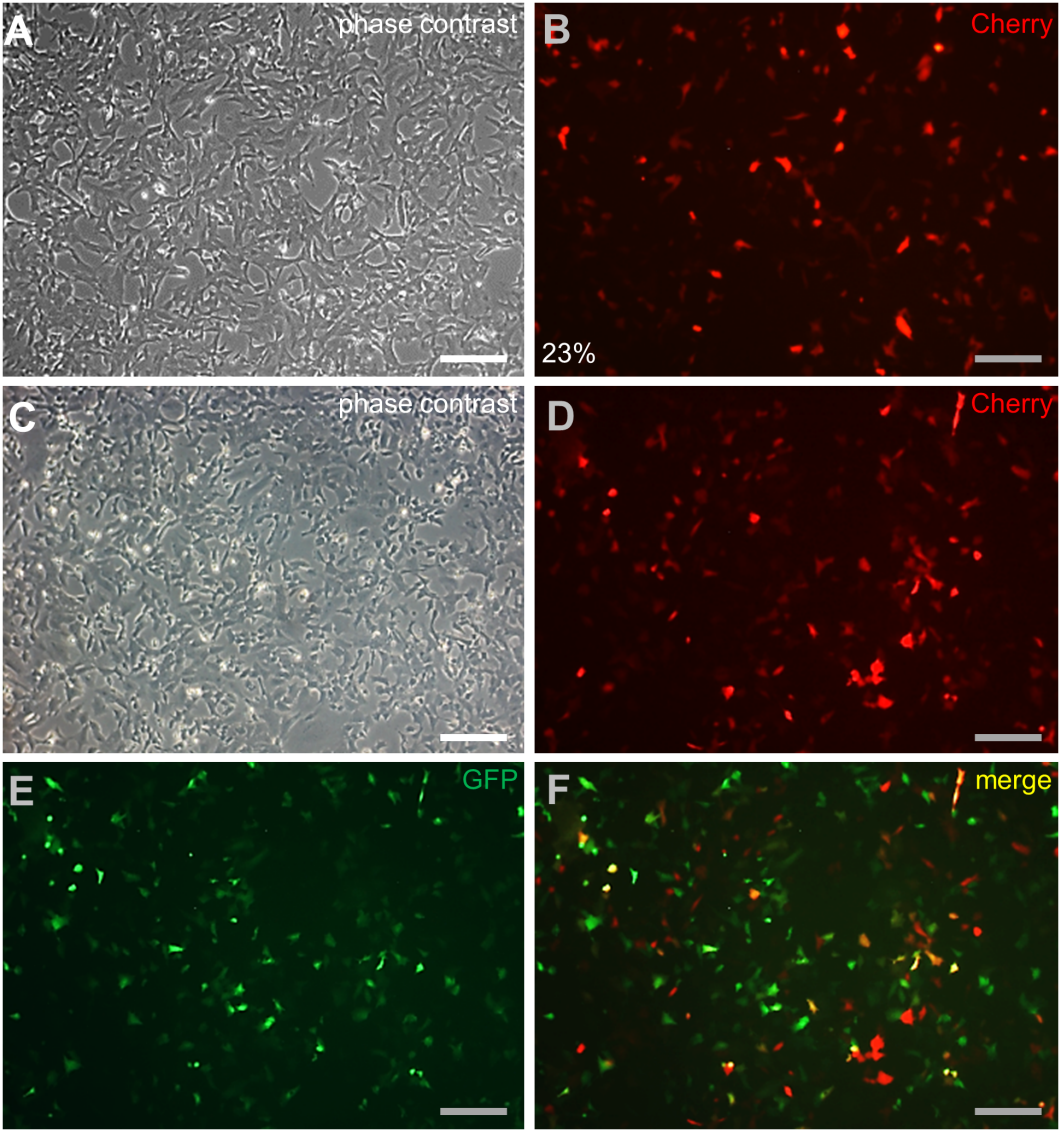
Cotransduction of SG3 cells. Medaka spermatogonia cells SG3 were infected with rvLcherry alone or plus rvLegfp at MOI of 50 and photographed at 3 dpi. (A and B) SG3 after rvLcherry infection. (C-F) SG3 after infection with rvLcheery and rvLegfp. Scale bars, 100 μm.

We wanted to determine cotransduction by using rvLegfp and rvLcherry. To this end, SG3 was chosen because of its highest transduction efficiency among fish cells tested. Infection with rvLcherry at MOI = 50 produced Cherry-positive cells at 23% efficiency (Fig 3A and 3B), the same as that of rvLegfp in this cell line (see Fig 1E). When SG3 was coinfected with rvLcherry and rvLegfp at MOI = 50 and monitored for transgenic reporter expression at 3 dpi, roughly equal numbers of cells were found to be positive for Cherry and GFP (Fig 3C–3F). These results suggest that rvLcherry and rvLegfp are equally proficient for SG3 transduction and detection by fluorescent microscopy.

### Gene trapping in medaka haploid ES cells

**Fig 4:**
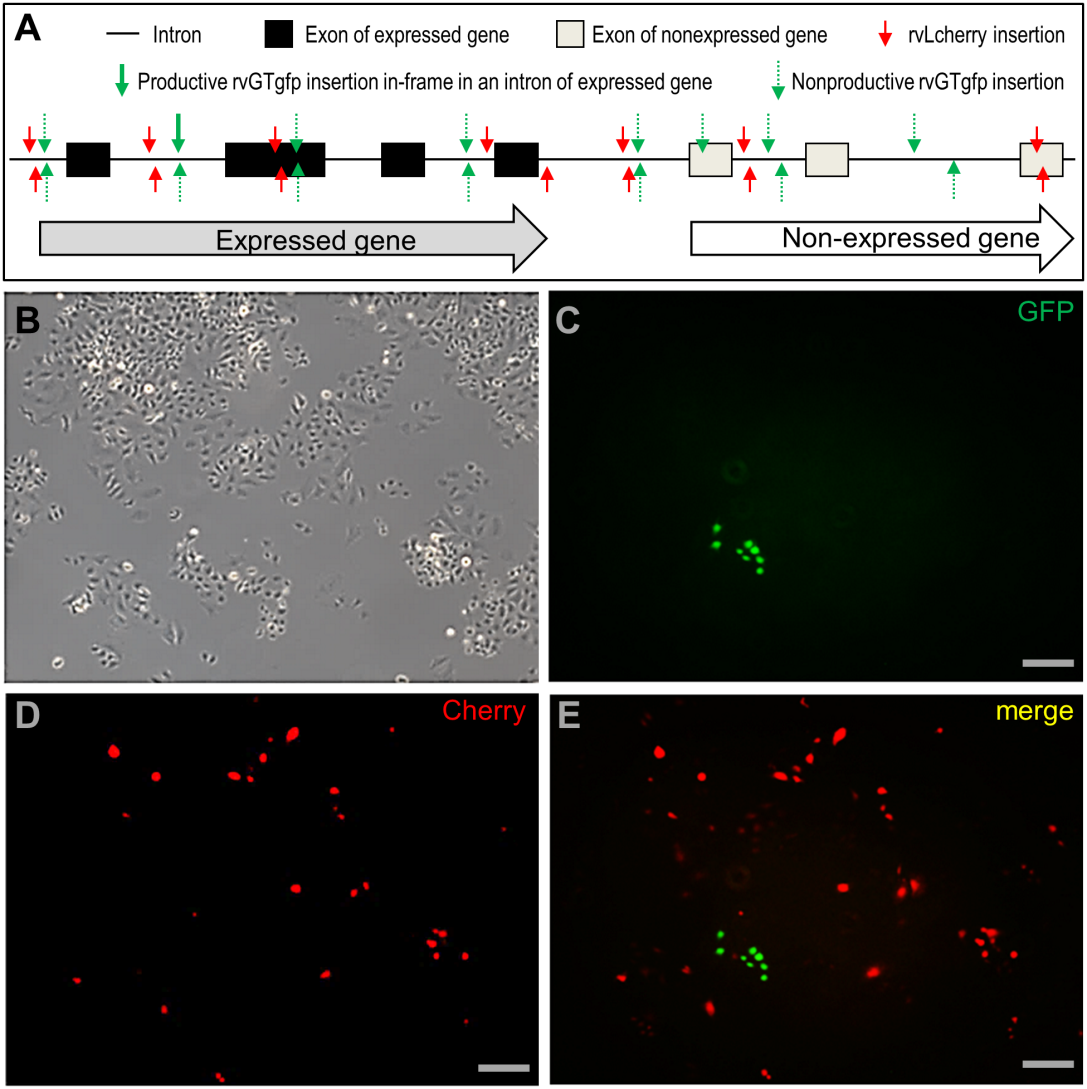
Gene trapping in haploid medaka ES cell line HX1. Medaka haploid ES cell line HX1 was infected with rvLcherry and rvGTgfp at MOI = 50 each and observed for gene trapping (green) and random gene insertion (red) at 3 dpi. (A) Consequence of retroviral integration on reporter expression. Any insertions by rvLcherry are predicted to be productive for Cherry expression due to presence of the strong promoter CV. Only a minority of insertions by rvGTgfp is productive for GFP expression due to absence of a promoter. For more details on reporter expression cassettes see Fig 1A. (B) Phase contrast micrograph, showing cell density and phenotype. (C) Cherry micrograph, showing Cheery expression from random insertions. (D) GFP micrograph, showing GFP expression from gene trapping. (E) Merged fluorescent micrograph. Scale bars, 100 μm.

In both rvLcherry and rvLegfp, the reporter gene cherry or egfp is under the control of a strong promoter. Upon infection, they can express the reporter at any random chromosomal sites (red arrows; Fig 4A). By contrast, the gfp in rvGTgfp is linked to a consensus splicing acceptor (SA) but lacks a promoter. Consequently, rvGTgfp will acquire the ability to express GFP only upon productive gene trapping, namely the in-frame integration into an intron in the same orientation as the transcription direction of an endogenously expressed gene (solid green arrows in Fig 4A) to produce a fusion protein (S3 Fig), whereas other insertions will be non-productive for reporter expression (broken green arrows; Fig 4A). As a result, rvGTgfp would produce fewer GFP-positive cells than Cherry-positive cells by rvLcherry in coinfection experiments. To test this, the haploid ES cell line HX1 was used for coinfection and monitored for reporter expression. Coinfection did not alter cell growth (S4 Fig) and phenotype (Fig 4B), produced many Cherry-positive cells arisen from random integrations and, as expected, fewer GFP-positive cells arisen from gene trapping events (Fig 4C–4E).Taken together, RVs are proficient for mediating random chromosomal insertions including trapping of endogenously expressed genes in medaka haploid ES cells.

### Genome-wide insertional mutagenesis

**Fig 5:**
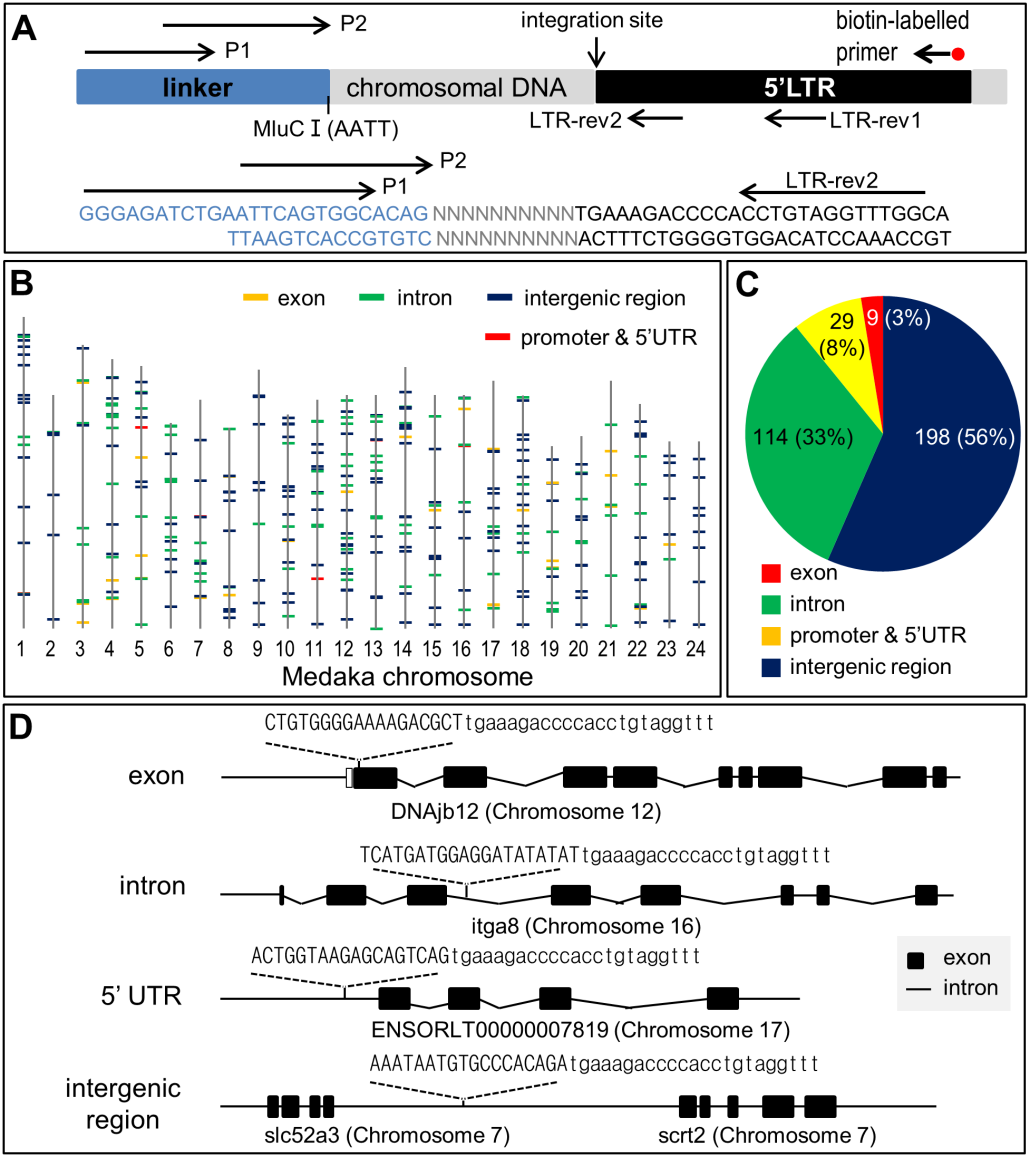
Genome-wide distribution of retroviral insertions. (A) Cloning and sequencing of insertion events by LM-PCR. MlucI digests of genome DNA was amplified by 5′-biotin-labeled primer for synthesis of ssDNA. The purified ssDNA was ligated with linker and subsequently amplified via two rounds PCR by using primers P1 plus LTR-rev1 and P2 plus LTR-rev2 and cloned for sequencing. (B) Chromosomal distribution of the 350 insertion events. vertical line, chromosome; horizontal bar, site of integration; color; genomic location. (C) Percent genomic locations of 350 insertion events. (D) Junction sequences of representative insertion events.

We then wanted to determine the precise sites of RV insertion relative to individual chromosomal genes. To this end, genomic DNA was isolated from rvGTgfp-transduced HX1 cells at 3 dpi and subjected to amplification by linker-mediated PCR (Fig 5A). Sequencing of cloned PCR products led to the identification of 350 insertion events. Sequence analyses revealed that insertion events distributed over all the 24 chromosomes of the haploid medaka genome (Fig 5B), and occurred at frequencies of 56%, 33%, 8% and 3% in intergenic regions, introns, promoters and 5’-UTRs, and exons, respectively (Fig 5C). Furthermore, the pGTgfp transgene was found to be inserted at an equal frequency in both forward and reverse orientations relative to the transcriptional direction of chromosomal genes. Representative insertion events are illustrated in Fig 5D. All of the intronic and exonic integration events might resulte in insertional mutations of genes concerned. Taken together, RV transduction can mediate genome-wide IM in medaka haploid ES cells.

### Retention of ES cell property

**Fig 6:**
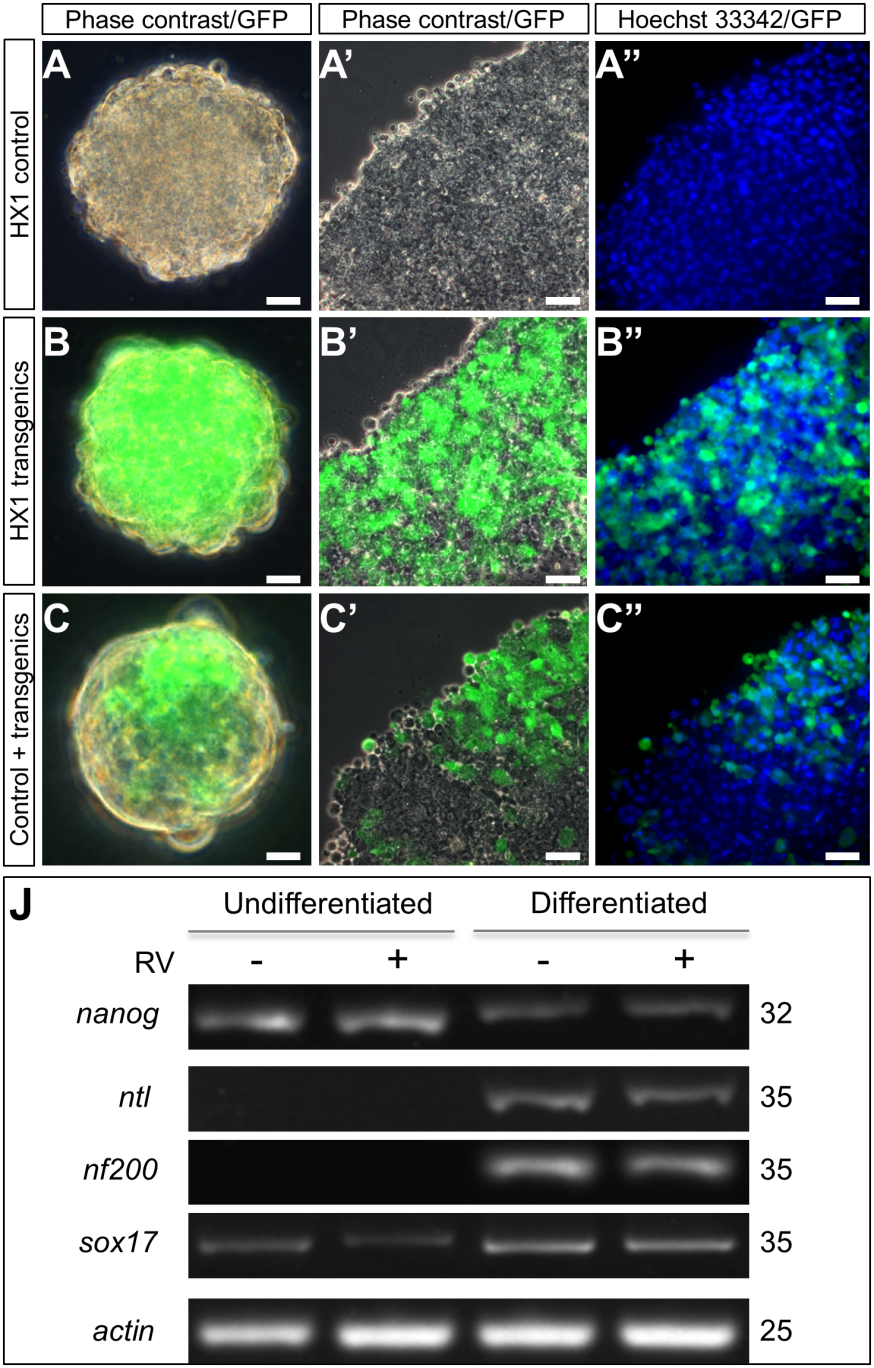
Retention of ES cell property. Pure populations of GFP-expressing transgenic HX1 cells were obtained by derived rvGTgfp transduction and FACS. Cells in three groups (HX1 control, HX1 transgenics and control + transgenics at a ratio of 1:1) were induced differentiation by EB formation maintained in suspension culture. EBs were directly photographed or stained with hoechst33342, squashed in slide and observed. transgenic HX1 cells(green), parental HX1 (unlabeled). Scale bar, 100 μm (Fig A, B, C), 25 μm (Fig A’, A”, B’, B”, C’, C”). (A, B, C) Three groups of cells were able to form typical EBs. Ebs were photograhped under phase contrast and GFP. (A’, B’, C’) EBs squashes under closer inspection of phase contrast and GFP. (A”, B”, C”) EBs squashes under closer observation of hoechst33342 and GFP. (J) Expression of pluripotency gene and differentiation genes in parental HX1 and transgenic HX1 before and after induced differentiation. Numbers of PCR cycles are indicated to the right. Genes chosen are markers for pluripotency (nanog) and differentiated lineages (nf200, ectoderm; ntl, mesoderm; sox17, endoderm). β-actin served as a loading control.

We wanted to determine whether RV-mediated transduction would have little adverse effect on the cellular property. To this end, we chose HX1 as a representative of the six cell lines. As a haploid ES cell line, HX1 is able to form a unique spherical structure called embryoid body (EB) in suspension culture and features expression pluripotency genes such as nanog and the ability for induced differentiation upon EB formation. Pure populations of GFP-expressing transgenic HX1 cells were obtained by derived rvGTgfp transduction and FACS. Parental HX1 control and HX1 transgenics were subjected to suspension culture in the presence of retinoic acid. HX1 formed typical EBs (Fig 6A). More importantly, GFP-expressing transgenic HX1 cells were able to form EBs under the same conditions (Fig 6B). In addition, when HX1 control and HX transgenics were co-cultured, they formed chimeric EBs (Fig 6C). A closer inspection on EB squashes clearly revealed the absence of any detectable difference in EB formation and cellular morphology between HX1 and its transgenic derivatives (Fig 6B’ and 6C”). An RT-PCR analysis revealed that HX1 transgenics were not different from HX1 control in gene expression profile before and after induced differentiation. Taken together, these results suggest that RV-mediated transduction does not compromise or alter the cellular property such as EB formation and gene expression profile.

## Discussion

In this study we have developed vector systems and procedure for RV-mediated stable gene transfer in fish stem cells and differentiated cells. We show that RV mediates a high efficiency of 11~23% for gene delivery and chromosomal integration in these fish cells. In the medaka ES cell line MES1, varying efficiencies of gene transfer have previously been produced by using different reagents and procedures. Specifically, Fugene, GeneJuice and electroporation resulted in efficiency of ~10%, ~20% and ~30% for plasmid DNA transfection [8], whereas a baculovirus system gave rise to an efficiency of more than 90% [7]. In these experiments, a transgene was frequently transferred transiently without chromosomal integration, and the actual efficiency of stable gene transfer remained unknown. In this study, although an efficiency of 11% obtained in MES1 by RV transduction is smaller than that of 3T3 probably due to CMV promoter expressing at lower efficiencies in zebrafish or medaka, it still represents the value for stable gene transfer, as RV-mediated transgene expression relies on chromosomal integration. Thus, RV appears to be the most efficient system for gene transfer and integration in fish cells.

The cell lines used in this study are different in origin, chromosomal ploidy level and differentiation status. These include the diploid ES cell lines MES1 and Z428 from midblastulae of medaka and zebrafish [41, 50], the haploid ES cell lineHX1 from medaka midblastulae [10], the diploid male germ stem cell line SG3 from the testicular spermatognia of the adult medaka [9], and differentiated fibroblast culture from the medaka fin. All of these cell lines show a similarly high efficiency for transduction by RVs, which is in accordance with the reported ability of RVs for transduction in a broad range of dividing cell types.

In human near-haploid leukemia cells of KBM7 line, RV-mediated gene insertion occurs at frequencies of 45%, 50.2%, 3.5% and 1.4% in intergenic regions, introns, promoters and 5’-UTRs, and exons, respectively[51]. These values obtained in this study are 56%, 33%, 8% and 3% for medaka haploid ES cells. These data suggest that fish and human cells have a generally similar tendency of RV-mediated gene insertion predominantly in intergenic and intron sequences. A recent study has reported a new form of MLV-based vectors which target away from intergenic and intron sequences [52]. Whether this approach is useful in fish remains to be determined in the future.

Previously, we have shown that plasmid transfection and drug selection do not compromise the medaka diploid ES cell property [8]. In this study, we show that stable gene transfer by RV transduction in medaka haploid ES cells also lead to the retention of the ES cell property, as pure populations of RV-transduced HX1 cells are not different from their parental cells in all of the three key parameters characteristic of the ES cell property, namely stable growth, embryoid body formation and gene expression profile.

Genetic screens by RV-mediated IM in haploid cell lines have widely been used for the identification of genes essential for host factors required by bacterial and viral infection [33–37] and for cellular phenotypes [38–40].The availability of haploid ES cell lines makes medaka a unique model organism of lower vertebrates for genetic screens. Recently, medaka haploid ES cell lines have been reported to be susceptible for infection by DNA and RNA viruses of aquaculture-important fish species [49]. Meanwhile, the current results show that RV mediates a high efficacy of gene transfer and genome-wide IM in medaka haploid ES cells. These results demonstrate the feasibility to make use of medaka haploid ES cells for IM-mediated genetic screens towards the identification of cellular genes essential for viral infection and other cellular processes such as pluripotency maintenance and cell differentiation.

## Supporting Information

S1 Fig Retroviral transduction in zebrafish ES-like cells. Z428 was infected with rvLegfp at MOI = 50 and photographed at 3 dpi. (Phase contrast micrograph showing cell density and phenotype. (B) Fluorescent micrograph showing transgenic GFP expression. Average percentage of GFP-positive cells (green) derived by cell counting in three independent experiments is shown to the left corner. Scale bars, 100 μm.
(TIF)

S2 Fig Retroviral gene trapping in the medaka haploid ES cell line HX2. HX2 was transduced by pGT-gfp and pLcherry at MOI = 50 each and observed at 3 dpi for gene-trap (green) and random gene insertion (red). Scale bars, 100 μm.
(TIF)

S3 Fig RV-mediated gene trapping and transgene expression. Gene trapping cassette GTgfp contains the SA-gfp-polyA flanked by two LTRs. After proper integration into an actively expressed gene, GFP expression will be driven by the endogenous promoter of that chromosomal gene. An in-frame integration will generate a fusion transcript for a fusion protein that contains only the N-terminal part but lacks the remainder of the encoded protein, thus leading to the mutation of the chromosomal gene. SA, splice acceptor sequence; LTR, Long terminal repeat; GFP, green fluorescent protein.
(TIF)

S4 Fig Growth curve. Similar doubling time (Td) is detected between rvLcherry and rvGTgfp co-infected HX1 cells (A) and parental HX1 cells (B).
(TIF)

S1 Table Genes and primers used for RT-PCR. (DOCX)
