# Supplementary figures and images for: Gene Transfer and Genome-Wide Insertional Mutagenesis by Retroviral Transduction in Fish Stem Cells
Source: PLoS One. 2015 Jun 1;10(6):e0127961. doi: 10.1371/journal.pone.0127961 (PMC4451014; doi:10.1371/journal.pone.0127961)

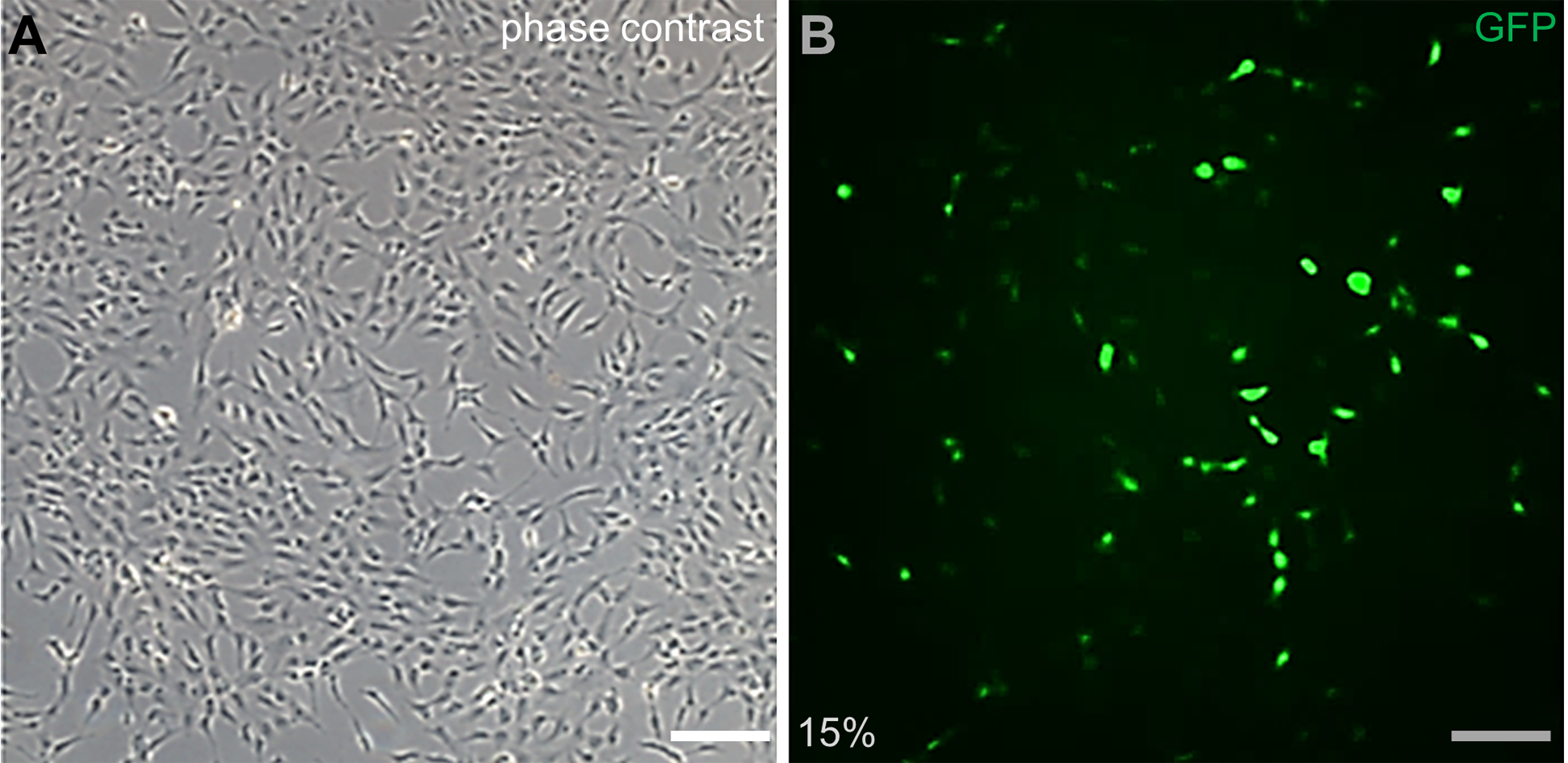

Supplement: S1 Fig — Z428 was infected with rvLegfp at MOI = 50 and photographed at 3 dpi. (Phase contrast micrograph showing cell density and phenotype. (B) Fluorescent micrograph showing transgenic GFP expression. Average percentage of GFP-positive cells (green) derived by cell counting in three independent experiments is shown to the left corner. Scale bars, 100 μm. (TIF) [file pone.0127961.s001.tif]

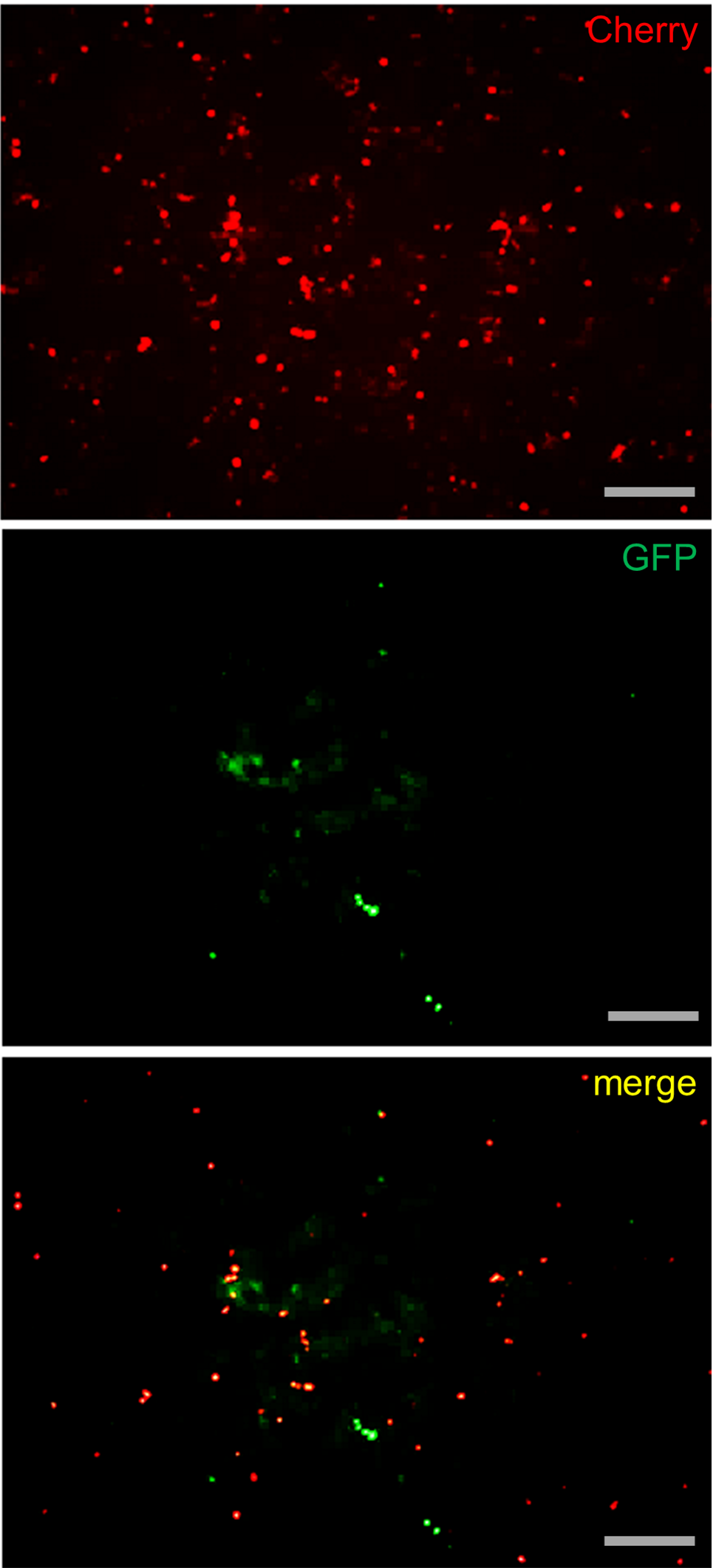

Supplement: S2 Fig — HX2 was transduced by pGT-gfp and pLcherry at MOI = 50 each and observed at 3 dpi for gene-trap (green) and random gene insertion (red). Scale bars, 100 μm. (TIF) [file pone.0127961.s002.tif]

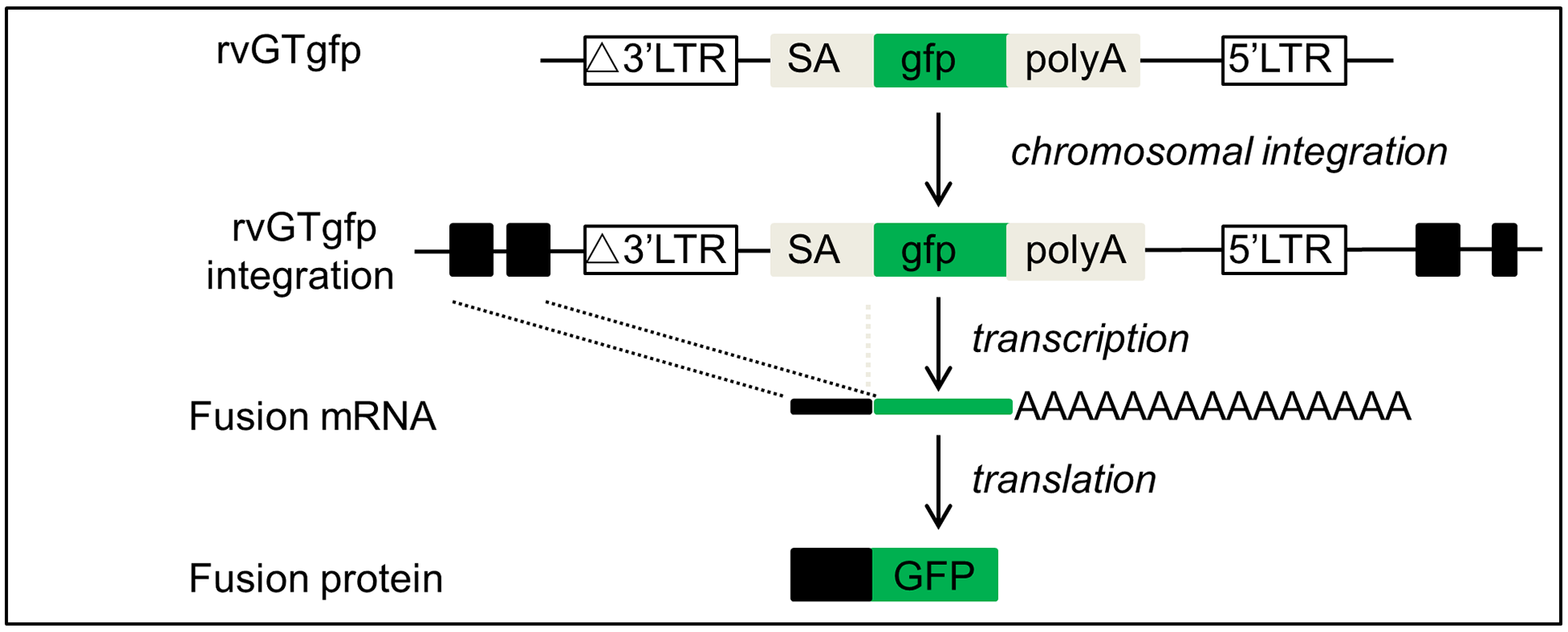

Supplement: S3 Fig — Gene trapping cassette GTgfp contains the SA-gfp-polyA flanked by two LTRs. After proper integration into an actively expressed gene, GFP expression will be driven by the endogenous promoter of that chromosomal gene. An in-frame integration will generate a fusion transcript for a fusion protein that contains only the N-terminal part but lacks the remainder of the encoded protein, thus leading to the mutation of the chromosomal gene. SA, splice acceptor sequence; LTR, Long terminal repeat; GFP, green fluorescent protein. (TIF) [file pone.0127961.s003.tif]

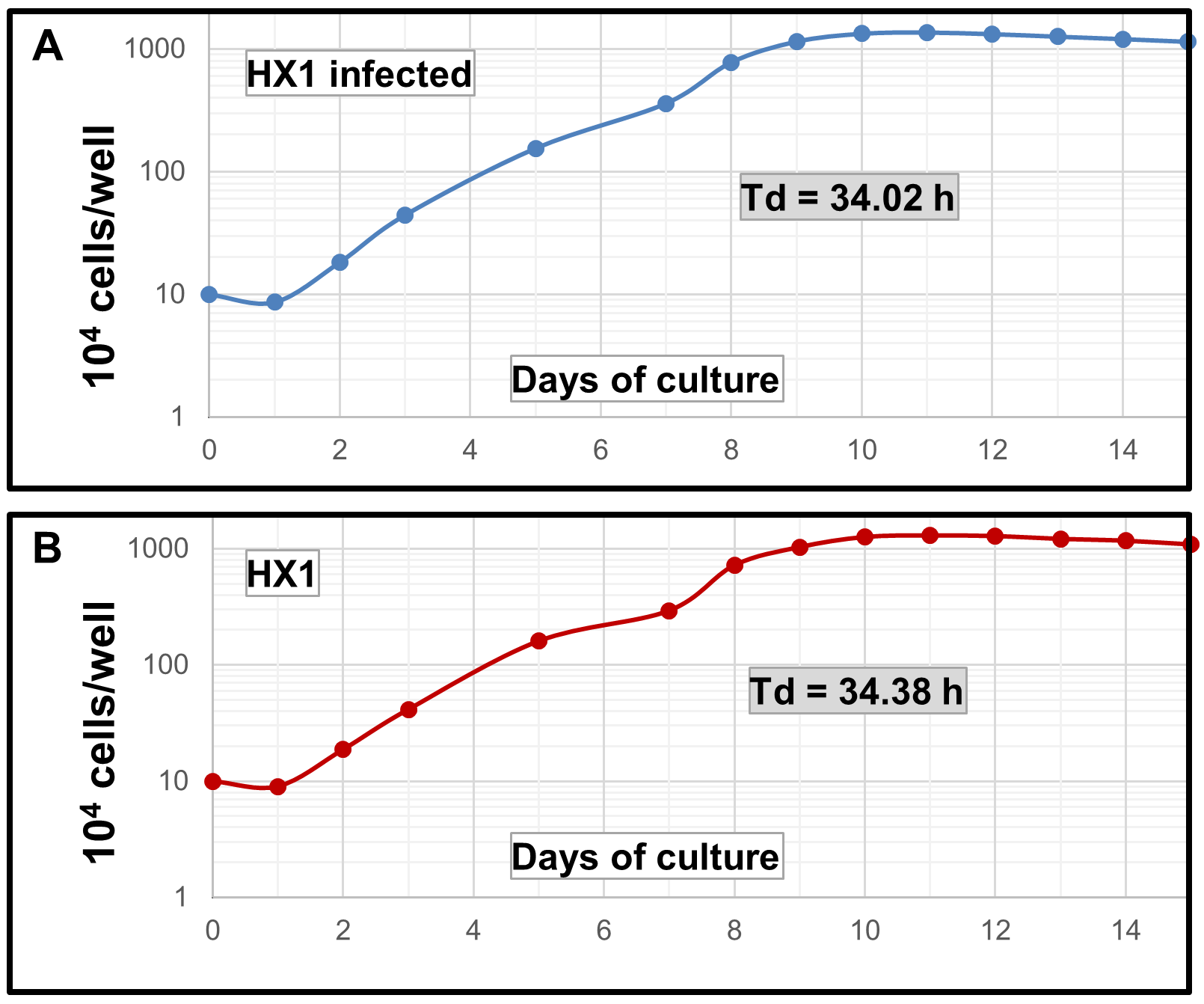

Supplement: S4 Fig — Similar doubling time (Td) is detected between rvLcherry and rvGTgfp co-infected HX1 cells (A) and parental HX1 cells (B). (TIF) [file pone.0127961.s004.tif]
