# Supplementary material for: Gene Transfer and Genome-Wide Insertional Mutagenesis by Retroviral Transduction in Fish Stem Cells
Source: PLoS One. 2015 Jun 1;10(6):e0127961. doi: 10.1371/journal.pone.0127961 (PMC4451014; doi:10.1371/journal.pone.0127961)
Supplement: S1 Table — (DOCX) [file pone.0127961.s005.docx]

**Table. S1. Genes and primers used for RT-PCR**

| Category | Gene (accession) | Primers (5’ to 3’) | Size( bp) |
| --- | --- | --- | --- |
| Stemness | nanog (NP_001153902) | nanog F:ATGGTTGAGTCCCAATCTTTTG  nanog R:TCAATATCGCTCTGAAACCCAG | 324 |
| Ectoderm | nf200 (ENSG00000100285) | Nf200 F:AGAAGAACCACGAGGAAGAAGT  nf200 R:GAGAGTCGGCTATCCAAGTAGG | 577 |
| Mesoderm | brachyury (ntl, no tail) (ENSORLG00000011262) | ntl F:ATGAGCGCGTCGAACCCGGAC  ntl R:AGACGGGCGCTTTCATCCAGT | 403 |
| Endoderm | sox17 (ENSORLG00000011542) | sox17 F:TGATGCCTGGAATTGGACACT  sox17 R:GCTGCGAAGGAACCTGGAA | 504 |
| House keep | actin (D89627) | actin F: CATGTGCAAAGCCGGATTCG  actin R:GTAGTCTGTAAGGTCGCGGC | 520 |
